# Pharmacy refill data can be used to predict virologic failure for patients on antiretroviral therapy in Brazil

**DOI:** 10.7448/IAS.20.1.21405

**Published:** 2017-06-05

**Authors:** David Martin, Paula M. Luz, Jordan E. Lake, Jesse L. Clark, Dayse P. Campos, Valdilea G. Veloso, Ronaldo I. Moreira, Sandra W. Cardoso, Jeffrey D. Klausner, Beatriz Grinsztejn

**Affiliations:** ^a^ Department of Emergency Medicine, Highland Hospital, Oakland, CA, USA; ^b^ Instituto Nacional de Infectologia (INI), Fundacao Oswaldo Cruz, Rio de Janeiro, Brazil; ^c^ Department of Medicine, University of California, Los Angeles, Los Angeles, CA, USA

**Keywords:** adherence, antiretroviral therapy, pharmacy refill, cohort studies, Brazil, viral load

## Abstract

**Introduction**: Pharmacy adherence measures such as pharmacy dispensing ratios (PDRs) have previously been shown to be predictive of virologic outcomes. We aimed to determine the optimal interval of PDR assessment for predicting virologic failure for HIV-infected patients on antiretroviral therapy (ART).

**Methods**: Using national Brazilian ART pharmacy refill data, we examined PDRs for patients ≥18 years of age with at least one HIV RNA level ≥180 days after ART initiation on or after 1 January 2011. Patients with a documented ART change ≤270 days prior to viral load test date were excluded. Logistic regression models were used to describe associations between virologic failure, defined as an HIV RNA level ≥400 copies/mL and PDRs, defined as the number of days index drug dispensed (non-nucleoside reverse-transcriptase inhibitor or protease inhibitor) per 180- and 90-day, interval preceding viral load testing, adjusting for sex, age, race, time since ART initiation and index drug. Backward elimination of insignificant variables was performed after adjusting for PDR. A predictive probability of virologic failure was calculated using the corresponding odds ratios for the PDR and any other significant variables. The diagnostic performance of the PDR interval was assessed by calculating the area under the receiver operating characteristic curve (AUROC) for the predictive probability with respect to virologic failure.

**Results and Discussion**: A total of 1,025 patients were included (68% were male, median age 40 years, median time on ART 3.4 years). The PDR was found to be significantly associated with virologic failure for all of the PDR intervals (*p* < 0.001). There was an increased risk of virologic failure for all PDRs <0.95. The 90–180 days interval had a AUROC of 0.842, compared to 0.841 and 0.829 for the 0–180 days and 0–90 days intervals, respectively. The PDR performed well as a predictive tool to identify patients in virologic failure with the 90–180-days interval prior to viral load testing being marginally more predictive.

**Conclusions**: The validation and use of the pharmacy dispensing ratio using public pharmacy refill data could aid in early identification of patients with poor adherence and prevent development of treatment failure and drug resistance in Brazil.

## Introduction

The ability to achieve and maintain viral load suppression for human immunodeficiency virus (HIV)-infected patients on antiretroviral therapy (ART) is dependent on, among other factors, a high level of adherence to the drug regimen [1–3]. Suboptimal adherence to ART has been shown to reduce viral load suppression [4] and increase the risk of viral resistance [5] and death [6]. Previous studies have demonstrated significant differences in viral load suppression for patients whose treatment adherence was ≤95% [2,3].

Given the importance of adherence to ART in patient outcomes, various methodologies exist for assessing levels of adherence to ART, including patient self-report, pharmacy-based measures, pill-counts, and biological markers [7]. Patient self-reports have been shown to be specific, but not sensitive to non-adherence and can only assess a limited period due to limitations in recall [8]. Pharmacy adherence measures, unlike patient self-reports, are objective measures of adherence which can be obtained from clinic records, and are therefore not subject to patient recall and social desirability bias [9]. However, pharmacy measures serve only as estimates of maximum adherence because they do not take into account individual pill-taking behaviour [10]. Pill counts and biological markers are resource-intensive strategies and therefore are not likely to be cost-effective strategies for use in resource-limited settings [10].

Studies have demonstrated the association of various measures of adherence and plasma HIV RNA levels [2,11,12]. Interview collected adherence levels have been previously shown to be predictive of viral load suppression, but not to the extent to substitute for routine viral load testing. Pharmacy measures have consistently been shown to predict current virologic outcomes; however, additional studies are needed to define the best parameters of pharmacy measures that are most predictive of future clinical and virologic outcomes, and best suited for use in clinical practice [10]. This study assessed the validity of using pharmacy dispensing ratios (PDRs) for predicting the likelihood of virologic failure for patients on ART, as well as determine the most predictive assessment interval for PDRs within our clinical cohort.

## Methods

### Study population

We conducted a cross-sectional study using data obtained from the longitudinal, clinical database of HIV-infected patients receiving care at the Instituto de Pesquisa Clinica Evandro Chagas (IPEC) in Rio de Janeiro, Brazil. IPEC has been providing care to HIV-infected patients since 1986. The clinical database was established in 1998 and has since been updated regularly using outpatient and inpatient medical records, and laboratory results. Pharmacy prescription pickup data were obtained from the national Brazilian ART pharmacy control system. That database contains information regarding drug regimen, number of days' supply of medication dispensed, regimen changes and justification for drug regimen changes for all patients enrolled in the National HIV/AIDS Programme.

Patients greater than or equal to 18 years of age with at least one HIV RNA level occurring from 1 January 2011 through 31 December 2011 greater than or equal to 180 days after ART initiation to allow time for virologic response to treatment were included in the analysis. Patients with a documented regimen change less than or equal to 270 days prior to viral load test date were excluded to ensure patients were on a stable regimen at time of viral load testing and to allow for virologic response to treatment changes.

### Variables of interest

The first HIV RNA viral load test occurring greater than or equal 180 days after ART initiation occurring from 1 January 2011 through 31 December 2011 was analyzed for all patients. The outcome of interest was virologic failure, defined as ≥400 copies/mL, to account for less sensitive virus detection thresholds of older testing platforms.

The PDR was calculated based on the number of days’ supply of index drug dispensed per 180-, and 90-day intervals preceding the viral load test date. A total of three assessment intervals were included; 0–180 days, 90–180 days and 0–90 days. Periods shorter than 90 days were not considered given that patients are generally required to pick up ARVs on a monthly basis. The index drug was defined as the non-backbone ARV included in the drug regimen, consisting of either a non-nucleoside reverse-transcriptase inhibitor (NNRTI) or protease inhibitor (PI). The decision to include only NNRTI- or PI-based regimens was based on prior data showing 90% of patients in treatment in 2011 were either on an NNRTI or PI based regimen [13]. No distinction was made between patients on first- or second-line regimens. The date of the last drug prescription dispensed within the assessment interval prior to the viral load test date was used to calculate the number of days left within the interval. If the number of days left within the interval was less than the days’ supply of the prescription, only the supply intended to be used within the interval was included in the calculation of the PDR to avoid overestimation of the PDR. The date of the first prescription dispensed prior to the start of the assessment interval was used to determine if any of the supply of drug dispensed prior to the interval was to be included in the calculation of the PDR to avoid underestimation of the PDR. For example, if a patient filled a 30-day prescription 5 days prior to the end of the interval, only the 5-day supply of drug intended to be used during the interval would be included in the calculation of the PDR. This method only accounts for overestimation based on the last prescription filled, therefore patients could theoretically be in possession of a greater supply of drug than the number of days within the period.

### Statistical methods

Adjusted logistic regression models were used to describe the association between virologic failure and PDR for each of the 180- and 90-day assessment intervals. The PDR was treated as a categorical variable according to the following thresholds, >0.95 or ≤0.95, which were based on prior studies citing increase likelihood of virologic failure in patients with ≤95% adherence [1,2]. Additional variables that were assessed as possible predictors of virologic failure included sex, age, race, time since ART initiation and index drug. Selection of variables was limited to information which was readily available in the  IPEC clinical database. Logistic regression models were used to test the associations between individual variables and virologic failure. All variables were included in the initial model and, assuming a significance level of p < 0.05, backward elimination of insignificant variables was performed after adjusting for PDR. The corresponding odds ratios (OR) for PDR and any additional variables found to be significant were used to calculate a predictive probability of virologic failure. The diagnostic performance of each PDR interval was assessed by calculating the area under the receiver operating characteristic curve (AUROC) for the predictive probability with respect to virologic failure. An optimal cut-point for the predictive probability was determined based on maximizing the sensitivity and specificity for predicting the likelihood of virologic failure. The cut-point therefore represents the predicted probability threshold above which a patient would be considered to be in virologic failure. The bootstrap method was then used to calculate the sensitivity and specificity and corresponding 95% confidence intervals of the predictive probability cut-point for each of the PDR intervals.

### Ethical approval

This study was approved by the ethics committee of the Evandro Chagas Clinical Research Institute of the Oswaldo Cruz Foundation (CAAE 0032.0.009.000–10). Written informed consent for enrolment in the cohort was obtained from all patients. Retrospective analysis of previously collected data delinked from personal identifying information was considered exempt by the Columbia and University of California Los Angeles IRBs.

## Results

### Characteristics of the study population

A total of 1,025 patients were included in the analysis of which 68% were male with a median age [interquartile range (IQR)] of 40 years [33, 47] and median time since ART initiation of 3.4 years [1.8, 6.0] of which 57% were on an NNRTI. Table 1 provides an overview of the demographic and clinical characteristics of the study population. Overall, 10.6 of patients were found to be in virologic failure. The median time since ART initiation in years [IQR] for patients found to be in virologic failure was 4.1 [2.3, 7.0] compared to 3.4 [1.8, 5.8] for patients not in virologic failure.

**Table 1.** Overall patient demographic and clinical characteristics

Characteristics	n = 1025
Sex	
Male: n (%)	699 (68)
Female: n (%)	326 (32)
Race	
White: n (%)	537 (52)
Non-White: n (%)	488 (48)
Age (yrs): median (IQR)	40 (33, 47)
Time since Initiation of ART (yrs): median (IQR)	3.4 (1.8, 6.0)
Regimen containing	
Efavirenz	586 (57)
Atazanavir	200 (20)
Lopinavir/Ritonavir	239 (23)
Virologic Failure: n (%)	109 (10.6)

Less than 33% of patients had a PDR >0.95 (Table 2). The median PDR was similar between the three interval categories. A small number of patients had a PDR>1.0. Table 2 provides a description of the PDR variable by assessment interval.

**Table 2.** Characteristics of Pharmacy Dispensing Ratios (PDRs) by assessment interval

Interval	Median [IQR]	PDR ≤0.95 (%)	PDR >0.95 (%)
0–180 Days	0.75 [0.62, 0.99]	71%	29%
90–180 Days	0.75 [0.58, 1.01]	74%	26%
0–90 Days	0.73 [0.58, 0.99]	67%	33%

IQR: interquartile range

### Predictors of virologic failure

The PDR was found to be significantly associated with virologic failure for all of the PDR intervals (Table 3). There was an increased risk of virologic failure for all PDRs <0.95. Of the additional variables tested, only race and index drug were significantly associated with virologic failure after adjusting for PDR. Sex, age and time since ART initiation were not found to be significantly associated with virologic failure. PI-based regimens were significantly associated with an increased risk of virologic failure when compared to an NNRTI-based regimen across all PDR intervals. Patients on lopinavir/ritonavir had a more than two-fold increase risk of virologic failure compared to those on atazanavir. Table 3 displays the results of the adjusted logistic regression models describing the association between PDR and virologic failure.

**Table 3.** Factors associated with virologic failure (≥400 Copies/mL)

	Intervals					
	0–180 Days		90–180 Days		0–90 Days	
Significant Variables	OR	P-value	OR	P-value	OR	P-value
PDR >0.95 (reference)	-	-	-	-	-	-
PDR ≤0.95	7.03	<0.001	6.00	<0.001	5.14	<0.001
White (reference)	-	-	-	-	-	-
Non-White	1.79	0.010	1.76	0.014	1.66	0.023
Efavirenz (reference)	-	-	-	-	-	-
Lopinavir/Ritonavir	12.33	<0.001	13.41	<0.001	12.67	<0.001
Atazanavir	5.05	<0.001	5.27	<0.001	5.12	<0.001

PDR: pharmacy dispensing ratio; OR, odds ratio

All of the PDR assessment intervals were found to have statistically significant AUROC. The 90–180 days interval was marginally superior at predicting virologic failure. Table 4 displays the diagnostic performance of the predictive probability and associated sensitivities and specificities for the selected predictive probability cut-points.

**Table 4.** Diagnostic performance of the predictive probability cut-point above which patients are considered to be in virologic failure

	0–180-day interval	90–180-day interval	0–90-day interval
AUROC [95% CI]	0.841 [0.806, 0.875]	0.842 [0.806, 0.877]	0.829 [0.791, 0.867]
Predictive probability cut-point	0.14	0.14	0.20
Sensitivity [95% CI]	83% [76%, 90%]	79% [71%, 87%]	73% [64%, 81%]
Specificity [95% CI]	74% [71%, 77%]	77% [74%, 80%]	78% [75%, 81%]

AUROC: area under the receiver operating characteristic curve; CI: confidence interval

## Discussion

In our observational clinical cohort, the PDR performed well as a predictive tool to identify patients in virologic failure. Our results are consistent with prior studies which demonstrate that the PDR can be used as a predictor of virologic failure and that upstream measures of adherence (>90 days prior to test date) are better predictors of virologic failure than more recent measures [10,14]. The predictive value of PDRs based on 90-day versus 180-day intervals was found to be similar with the most predictive interval being the 90-day period occurring 90–180 days prior to viral load testing.

We acknowledge that the sensitivities and specificities obtained are not sufficient to replace the need for viral load monitoring. However, our results demonstrate the possibility of developing a cost-effective predictive tool that could be used at the national, regional or district level by public health authorities to identify patients at highest risk of virologic failure, in settings where the cost of more expensive strategies such as pill counts and biological markers are prohibitive.

Sex, age and time since ART initiation were not significantly associated with virologic failure after adjusting for PDR. Non-White race was found to be associated with an increased risk of virologic failure. This difference may potentially be explained by other factors not assessed within our model such as education and socioeconomic status, which may be correlated with race. A recent study within our cohort did not find race to be significantly associated with the odds of achieving virologic suppression; however, the prior study also adjusted for educational level, which was found to be significant factor [13].

Index drug was significantly associated with virologic failure and patients on PI- versus NNRTI-based regimens were more likely to be in virologic failure. We did not distinguish patients based on first- or second-line regimen, however, based on the 2008 National HIV/AIDS Program treatment guidelines, NNRTI-based regimens are the preferred first-line regimen and lopinavir/ritonavir is preferred over atazanavir as the second-line PI [15]. Moreover, a prior study demonstrated that of the 1,311 patients initiating ART from January 2000 through June 2010 in our clinical cohort, 75.3% were started on a NNRTI-based regimen [16]. Therefore, one can assume that patients on PI-based regimens were more likely to represent patients who had failed first-line therapy, with poor adherence being one of the possible explanations for prior treatment failure. Differences in tolerability and dosing frequency may also account for the increased risk of virologic failure observed within patients on lopinavir/ritonavir compared to those on atazanavir [17,18]. Further discussion regarding the differences in the associations between index drug and virologic failure is beyond the scope of this report.

The generalizability of our study is limited because we derived the predictive model using only one cohort population. In order to validate further the national use of this predictive model, we would need to replicate our model using other patient databases within the national Brazilian ART pharmacy control system. Perhaps the greatest limitation of our study was the statistical programming required for the derivation of the predictive probability, which limits the ability to apply this tool to day-to-day clinical practice. However, the model could also be used as a surveillance tool by public health authorities to identify the geographic areas with the highest number of patients at risk of virologic failure, in order to implement targeted adherence interventions at the community level. In an effort to simplify the model, we calculated the PDR based solely on the index drug under the assumption that this would be a reliable proxy for overall adherence to therapy. We selected a threshold of >95% for the categorization of the PDR variable based on prior evidence supporting the use of this threshold; however, the median PDR for all intervals was only 0.75. Future research should evaluate lower PDR thresholds to determine if there is an optimal threshold for predicting virologic failure.

## Conclusions

In conclusion, we demonstrated the use of a predictive probability tool based on PDR and index drug for determining the likelihood of virologic failure at a future point in time for varying intervals of assessment. The validation and use of this predictive tool using pharmacy refill data available through the national Brazilian ART pharmacy control system could be of great benefit in early identification of patients with poor adherence and prevent development of treatment failure and drug resistance in Brazil. Following validation of these results, this tool could be applied at the national level in Brazil, and potentially other countries, to identify and further develop targeted adherence interventions in the geographic areas with the highest number of patients at risk of virologic failure. Additionally, further efforts should be made to explore the feasibility of designing a software program within the national Brazilian ART pharmacy control system that would automatize the calculation of the PDR and apply the predictive model on an ongoing basis, such that the system would automatically flag patients at highest risk of clinically significant non-adherence, thereby allowing clinical personnel to proactively intervene with targeted adherence interventions.
